# β-caryophyllene reduces inflammation to protect against ischemic stroke by suppressing HMGB1 signaling

**DOI:** 10.1186/s10020-025-01171-z

**Published:** 2025-03-24

**Authors:** Yuchun Wang, Yang Yang, Tuo Meng, Shengwei Liu, Jingdong Liu, Daohang Liu, Bharati Laxman, Sha Chen, Zhi Dong

**Affiliations:** 1 The Key Laboratory of Biochemistry and Molecular Pharmacology, Chongqing Medical University, Chongqing, 400016 China; 2 Department of Pharmacy, Chongqing Health Center for Women and Children, Women and Children’s Hospital of Chongqing Medical University, Chongqing, 401147 China; 3 Children’s Hospital of Chongqing Medical University, Department of Child Health in Children’s Hospital of Chongqing Medical University, Chongqing, People’s Republic of China; 4 Department of Pharmacy, Yongchuan Hospital of Chongqing Medical University, Chongqing, 402160 China

**Keywords:** Stroke ischemic stroke, Inflammatory, HMGB1, BCP

## Abstract

**Background:**

Ischemic stroke is characterized by high mortality and high disability rates and accounts for the vast majority of current stroke cases. Reperfusion after surgical treatment can cause serious secondary damage to ischemic stroke patients, but there are still no specific drugs for the clinical treatment of ischemic stroke. Inflammation plays a critical role in ischemia and reperfusion injury, highlighting the urgent need for new anti-inflammatory targets and therapeutic agents. High-mobility group box-1 (HMGB1) is highly expressed in both neuronal cell bodies and axons and has been found to have late proinflammatory effects; thus, the role of HMGB1 in stroke has recently become a hot research topic in critical care medicine. An increase in HMGB1 expression leads to the aggravation of inflammatory reactions after ischemic stroke. B-caryophyllene (BCP) is a natural drug with anti-inflammatory effects. However, whether HMGB1 is involved in the anti-inflammatory mechanism of BCP is still unknown. We aimed to investigate the relationship between HMGB1 and BCP in in vivo and in vitro ischemic stroke models.

**Methods:**

A middle cerebral artery embolism model was established in mice by thread thrombus, and primary neurons were subjected to oxygen‒glucose deprivation and reoxygenation (OGD/R) in vitro. In vitro, the HMGB1 DNA overexpression virus(GV-HMGB1)or the HMGB1 DNA silencing virus(RNAi-HMGB1)was injected into the lateral ventricles of mice..

**Results:**

HMGB1 expression increases after ischemic stroke and further affects the expression of TLR4, RAGE and other related inflammatory factors, thus reducing the inflammatory response and ultimately protecting against injury. These results confirmed the effect of HMGB1 on TLR4/RAGE signaling and the subsequent regulation of inflammation, oxidative stress and apoptosis. Furthermore, BCP potentially alleviates ischemic brain damage by suppressing HMGB1/TLR4/RAGE signaling, reducing the expression of IL-1β/IL-6/TNF-α, and inhibiting neuronal death and the inflammatory response.

**Conclusion:**

These data indicate that BCP exerts a protective effect against ischemic stroke-induced inflammatory injury by regulating the HMGB1/TLR4/RAGE signaling pathway, which provides new insights into the mechanisms of this therapeutic candidate for the treatment of ischemic stroke.

## Background

Stroke, including ischemic stroke and hemorrhagic stroke, is a local or global type of brain injury caused by complex factors, and ischemic stroke accounts for the vast majority of stroke cases (Ajoolabady et al. 2021). Since the 1990s, the incidence of stroke has increased annually, and the age of onset has decreased annually (Rosengren et al. 2013). In some countries, approximately 10% of all deaths are caused by a stroke (Goyal et al. 2015). With high morbidity and mortality, ischemic stroke can affect people of all ages, accounting for 88% of stroke cases (Fu et al. 2019). The lives of stroke patients and their families are seriously affected. At present, the main clinical treatment mode for stroke patients is surgery and drug treatment (Jovin et al. 2022). There is no specific drug for the treatment of ischemic stroke. Thrombolysis by surgery is usually used to treat acute ischemic stroke, which can lead to severe ischemia‒reperfusion injury. During ischemic stroke and reperfusion, injury leads to severe inflammatory reactions and neuronal death. Therefore, reducing the inflammatory response and protecting neurons has become important before and especially after thrombolysis in patients with ischemic stroke. New and more effective drugs need to be identified.

High-mobility group box-1 (HMGB1) is a nuclear protein with late proinflammatory effects (Zhang, et al. 2020) that is widely distributed in mammalian cells. Recent studies have shown that necrotic cells can release HMGB1 (Scaffidi et al. 2002; Zhao et al. 2023) to induce an inflammatory response. When damaged cells from HMGB1-/- mice were cultured with macrophages (Xue et al. 2020), the intensity of the inflammatory response was significantly reduced. The passive release of HMGB1 plays an important role in the tissue necrotizing inflammatory response. Reducing HMGB1 expression may be important for suppressing the inflammatory response in ischemic stroke.

β-Caryophyllene (BCP) is a kind of sesquiterpenoid that has strong anti-inflammatory activity and inhibits the inflammatory cascade (Agnes et al. 2023). Our previous studies revealed that BCP can reduce the volume of cerebral infarction after CIR injury in rats. However, the effect of BCP on HMGB1 expression in cerebral ischemia has not been studied.

The proinflammatory effect of HMGB1 is related to cell surface receptors, including Toll-like receptor (TLR4) and advanced glycosylation end product receptor (RAGE) (Yang et al. 2017). Bioinformatics analysis also revealed that the HMGB1, TLR4 and RAGE receptors are closely related or even directly related. Therefore, this study also aimed to explore whether there is a relationship between the levels of HMGB1 and downstream TLR4/RAGE activation. We hope to find potential drugs on this basis.

In this study, we explored the relationship between HMGB1/TLR4/RAGE and the effect of BCP in vivo and in vitro to provide a theoretical basis for the application of BCP in stroke.

## Methods

### Materials

Β-caryophyllene, 2,3,5-triphenyltetrazolium chloride (TTC), cytosine arabinoside (Ara-C), L-glutamine, and poly-L-lysine (0.1%) were purchased from Sigma (Sigma-Aldrich, St. Louis, MO, USA). All cell culture medium and fetal bovine serum (FBS) were obtained from GIBCO (Life Technologies, Grand Island, NY, USA). ELISA kit against interleukin-1b (IL-1b), interleukin-6 (IL-6), tumor necrosis factor-a (TNF-a) were obtained from USCN (Life Science Inc., Harrington Oakland,CA, USA). RNAi-HMGB1 and GV-HMGB1 were from Gene (Shanghai).

### Animals

Newborn C57BL/6 mice in 24 h and adult male mice C57BL/6 (20–25 g) in a specific pathogen-free (SPF) grade were obtained from the Experimental Animal Center, Chongqing Medical University (Chongqing, China). All animal procedures were approved by the Experimental Ethics Committee of Chongqing Medical University (Reference Number: 2015027) and performed in accordance with the National Institutes of Health Guide for the Care and Use of Laboratory Animals. All surgeries were performed under anesthesia, and all efforts were made to minimize the animals’ suffering.

### Primary neuron cultures

Primary cortical neurons were prepared from newborn mice (in 24 h) as described previously. Cortex were minced and dissected with trypsin–EDTA (0.125 mg/mL) in Hank’s balanced salt solution (HBSS). Neurons were cultured in plates, precoated with 0.01%poly-L-lysine, with Dulbecco’s Modified Eagle’s Medium medium containing 10% FBS. After 4 h of incubation, neurons were maintained in neurobasal A medium supplemented with 2% B27 and 1% glutamine (2 mM). Every 3 days, 50% of the culture medium was changed. Microtubule-associated protein-2 (MAP 2), the specific marker of neuron, was used to identify the purity of primary neurons by immunofluorescence. The primary cells, which commonly consist of > 95% neurons, were used in the experiments on the 7th day in vitro.

### Experimental design

The experimental design consists of three parts.

In the first part, the animals were divided into the sham group and the I/ R group, and the cells were divided into normal and OGD groups, to explore and confirm the occurrence of injury and the change of the key factors in the ischemic model.

In the second part, the animals were divided into four groups: sham group, I/R group, RNAi-HMGB1 group, GV-HMGB1 group. The cells were divided into four groups: normal group, OGD group, RNAi-HMGB1 group, GV-HMGB1 group. The RNAi-HMGB1 group refers to mice that underwent lateral ventricle injection of lentivirus to silence HMGB1 expression, while the GV-HMGB1 group refers to mice that received lateral ventricle injection of lentivirus to achieve HMGB1 overexpression. For the RNAi-HMGB1 group, cells were transfected with lentivirus to silence HMGB1 expression, while for the GV-HMGB1 group, cells were transfected with lentivirus to achieve HMGB1 overexpression.

The third part is to explore the relationship between BCP and HMGB1 pathway. The animals were divided into sham group, I/R group, and I/R BCP (72 mg/kg) groups, while the cells were divided into normal group, OGD group, and OGD BCP (10 μM) groups.

### Oxygen–glucose deprivation and re-oxygenation treatments in vitro/transient focal cerebral ischemia in vivo

Oxygen–glucose deprivation and re-oxygenation (OGD/R) were used as an in vitro model for ischemia (Zhang, et al. 2007). Briefly, at the seventh in vitro, neurons were washed and incubated with glucose-free medium, subsequently transferred to an anaerobic incubator equilibrated with 94% N2, 5% CO2, and 1% O2 at 37◦C for 1 h. The cells were then returned to the normoxic incubator with 25 mM glucose without serum for 24 h. Control neurons were cultured in the same medium supplemented with 25 mM glucose in a normoxic incubator.

Male mice underwent procedures to cause transient focal cerebral ischemia via right middle cerebral artery occlusion (MCAO) (Sugo, et al. 2002). Briefly, mice were anesthetized with isoflurane (induced with 3% and maintained by 1.0–1.5%) mixed with oxygen and nitrogen using a facemask. The right common carotid artery (CCA), internal carotid artery (ICA), and external carotid artery (ECA) were separated carefully under an operating microscope. A 6–0 nylon monofilament (Guangzhou Jialing Biotechnology Co., Ltd., Guangzhou, China) was inserted through the stump of ECA into the ICA and advanced into the middle cerebral artery until light resistance was felt (∼8–12 mm). After 1 h of MCAO, reperfusion was initiated by withdrawing the nylon monofilament. Sham-operated mice underwent identical procedure but the filament was not inserted. During the surgical procedure, rectal temperature was maintained at 37 ± 5  C using a thermostatically controlled infrared lamp. At 24 h of reperfusion, neurological function deficits were scored, and animals that scored from 1 to 4 were chosen for further experiment. Those animals that showed brain hemorrhage or with no ischemia (three mice) were exclude from the study. The mortality rate was 0.3%.

### BCP administration

Mice were given three days of continuous BCP (72 mg/kg) administration, and the body weight changes were recorded once a day. The fourth day, the model was established, the ischemia was 1 h, and the next experiment was carried out after 24 h of reperfusion.

BCP (10 nM) was given to primary nerve cells on the sixth day and a half after liquid exchange. 24 h after administration, OGD1 hours and reoxygenation 24 h later, the next experiment was continued.

### Detection of brain injury appearance

Neurological score–After 24 h of reperfusion, the neurobehavioral scores of mice in different treatment groups were evaluated by the Longa score (Longa, et al. 1989).

Cerebral infarct volume–The mice were given deep anesthesia after reperfusion for 24 h and decapitated for 15 min at -20 ℃, and then cut into 5 pieces (1 mm) and then incubated at 37 ℃ in 2% TTC staining solution for 30 min. The brain slices were put into 4% paraformaldehyde for 24 h, and transferred to 4% paraformaldehyde overnight. The brain slices was removed by filter paper and placed in a black background. The normal brain tissue was red after staining, and the area of the infarct was white. The digital camera was photographed and the volume of cerebral infarction was determined by image-Pro Plus 5 software.

Mice brains were infused with 4% neutral-buffered formaldehyde at indicated time, fixated for 24 h. Ethanol in graded concentrations and xylene were then used to dehydrate the brain tissue, and then they were embedded into paraffin. Hematoxylin and eosin (H&E) were used to stain the paraffin Sects. (5 μm), according to the standard protocol. Histological analysis of the same region in each experiment was performed with a light microscope. Paraffin sections were stained with toluene blue. The Nissan bodies in the same area were observed under light microscope.

### Immunohistochemical/immunofluorescence/Fluorescence probe in situ hybridization

Immunohistochemical–After paraffin section dewaxing, the antigen was repaired in microwave oven, and then the endogenous peroxide was blocked with 3% hydrogen peroxide. The sections were sealed with serum for half an hour, then the first antibody(HMGB1, 10,829–1-AP, Proteintech, 1:250) and the second antibody(Multi-rAb™ Polymer HRP-Goat Anti-Rabbit Recombinant Secondary Antibody (H + L) Cat No: RGAR011) were added, and then the nucleus was stained with DBA and restained with hematoxylin. After sealing the film, it was examined by microscope.

Immunofluorescence–After dewaxing, the antigen was repaired in microwave oven, the spontaneous fluorescence quenching agent was added, the serum was blocked for half an hour, and the first antibody and the second antibody were added respectively. Finally, the nucleus was restained with DAPI. After sealing the film, it was examined by microscope.

### Western blot analysis

Mouse ischemic brain tissues were harvested at 24 h post-reperfusion, and then homogenized in RIPA lysis buffer (P00113D; Beyotime, Shanghai, China). The whole ischemic brain tissues were used to determine HMGB1 and TLR4 and RAGE protein levels. The protein was separated using sodiumdodecyl sulfate–polyacrylamide gel electrophoresis (SDSPAGE; P0012A, Beyotime, China) with a 12% polyacrylamide gel and a 10% polyacrylamide gel, and then transferred to polyvinylidene fluoride (PVDF) membrane. Then the membranes were blocked with non-fat milk (5%) and incubated overnight at 4◦C with the following primary antibodies: rabbit polyclonal antibody against HMGB1 (10,829–1-AP, Proteintech, 1:250), TLR4 (19,811–1-AP, Proteintech, 1:250), RAGE (AF5309, Affinity, 1:1000), and mouse internal ginseng antibody (beta-actin; 10,829–1-AP, Proteintech, 1:1000). After three washes, secondary goat anti-rabbit/mouse (Bostor, China, 1:3,000) was performed to conjugate with alkaline phosphatase for 1 h at room temperature. Enhanced chemiluminescence was used to determine the immune reactivity. Gel imaging apparatus (Bio- Rad, Hercules, CA, USA) and Image Lab (Bio-Rad, Hercules, CA, USA) were used to scan and analyze the bands.

### PCR real time quantitative polymerase chain reaction analysis

Total RNA of cortex of ischemic brain were extracted using a Trizol kit (Sangon Biotech, Shanghai) and cDNA was prepared via using the AMV first chain cDNA synthesis kit (Sangon Biotech. Shanghai), according to manufacturer’s protocol. Real-time quantitative polymerase chain reaction (RT-qPCR) was performed in a 10 μL volume using SYBR Premix (Bimake). The following cycling conditions were used: 30 s at 95◦C followed by 40 cycles of 5 s at 95  C and 30 s at 60  C.

### Enzyme-linked immunosorbent assay (ELISA)

TNF-a, IL-1β, IL-6 levels in ischemic brain tissue homogenate were detected using an ELISA kit according the manufacturer’s instructions.

### Statistical analysis

All data are presented as the means ± SD. Graphpad software was used for statistical analysis. Differences between two groups were assessed by the t-test, differences between four groups were assessed by the ANOVA analysis and a value of P < 0.05 was considered statistically significant.

## Results

### Inflammation injury occurred and HMGB1 increased in mouse with MCAO

**Fig. 1:**
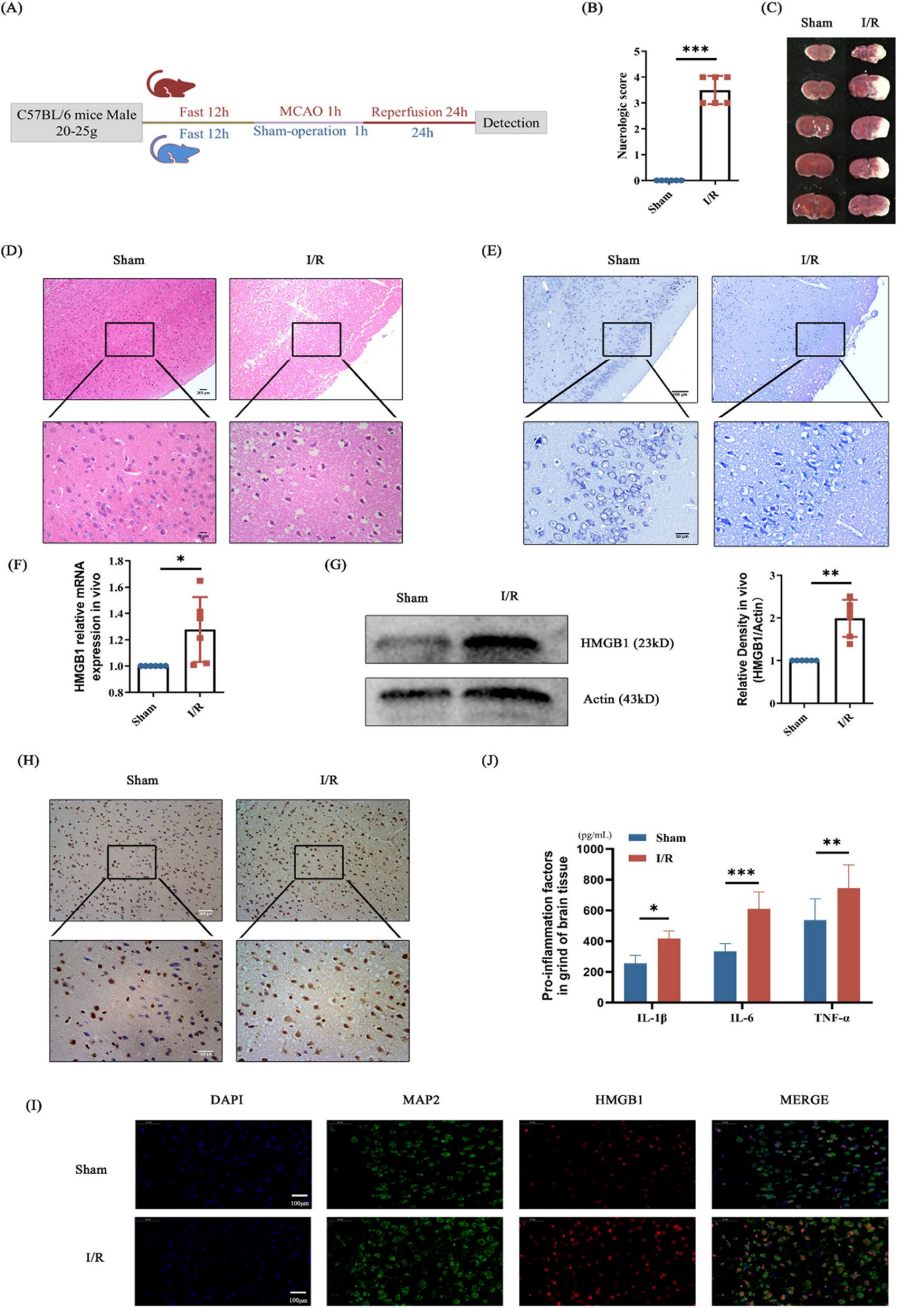
After cerebral ischemia and reperfusion, the mice developed brain damage, and the expression of HMGB1 was elevated. **A** Timeline of the mouse experiments. **B** Neurobehavioral evaluation of mice with or without MCAO. Higher scores indicate more severe neurological damage. **C** TTC staining of serial brain sections of the mice. White represents the infarcted fraction.** D** HE staining of the cortical portion of the mouse brain. Nuclei are colored in blue and the cytoplasm is colored in red. **E** Partial Nic staining of mouse cerebral cortex. The large and large number of Nissl bodies indicates that nerve cells have a strong function in protein synthesis; on the contrary, Nissl bodies will decrease or even disappear when nerve cells are damaged. **F** HMGB1 mRNA levels in ipsilateral brain tissues from control and MCAO mice. **G** Protein levels of HMGB1 relative to internal reference in ipsilateral brain tissue from control and MCAO mice. **H** Immunohistochemical staining of HMGB1 in ipsilateral brains of control mice and MCAO mice. Blue represents negative, and brown is positive. **I** Immunofluorescence staining of HMGB1 in the ipsilateral brain of control and MCAO mice. DAPI localized the nucleus and MAP 2 as a neural cell marker. **J** Detection of proinflammatory factors in ipsilateral brain tissue of control mice and MCAO mice. n = 6, * p < 0.05, ** p < 0.01, *** p < 0.001

Male mice were prematurely fasted for 12 h followed by right middle cerebral artery occlusion (MCAO) surgery, in which the sham group isolated vessels without plug line insertion (Fig. 1A). After an hour of clipping, the blood flow was restored. Mice status were examined 24 h later, and subsequent experiments were performed. The neurobehavioral score of mice with MCAO was higher than that of sham group (Fig. 1B). Consecutive brain sections stained with TTC indicate mouse with MCAO had cerebral infarction (Fig. 1C). HE staining showed injured brain tissue in MCAO group (Fig. 1D). Nissl staining showed that the injury of Nissl corpuscles was serious in the injury group (Fig. 1E). As can be seen from the above results, the MCAO model was successful, and the brain injury occurred. HMGB 1 has been shown to play a critical role in the development of late stages of inflammation (Zhang, et al. 2020). To further determine the mechanism of injury, we examined the expression of HMGB 1 after the onset of mouse brain injury. Brain tissues from mice were collected to detect level of HMGB1 in each group. The HMGB1 expression level in the brain was measured by western blot and Q-PCR assay. HMGB1 level was increased in injured group in vivo (Fig. 1F-G). The results of immunohistochemistry showed that HMGB1 was highly expressed in the injured group (Fig. 1H). The HMB1 expression level also measured by immune-fluorescence. It was found that the expression of HMGB1 raised sharply in I/R group compared with sham group (Fig. 1I). Finally, we examined the level of proinflammatory factors in mouse brain homogenate grinding fluid and found that the proinflammatory factor expression level was increased after MCAO surgery (Fig. 1J). At this point, we basically determined that inflammation occurred after cerebral ischemia and reperfusion, and HMGB 1 expression was elevated.

## Inflammation injury occurred and HMGB1 increased in primary neurons with OGD

**Fig. 2:**
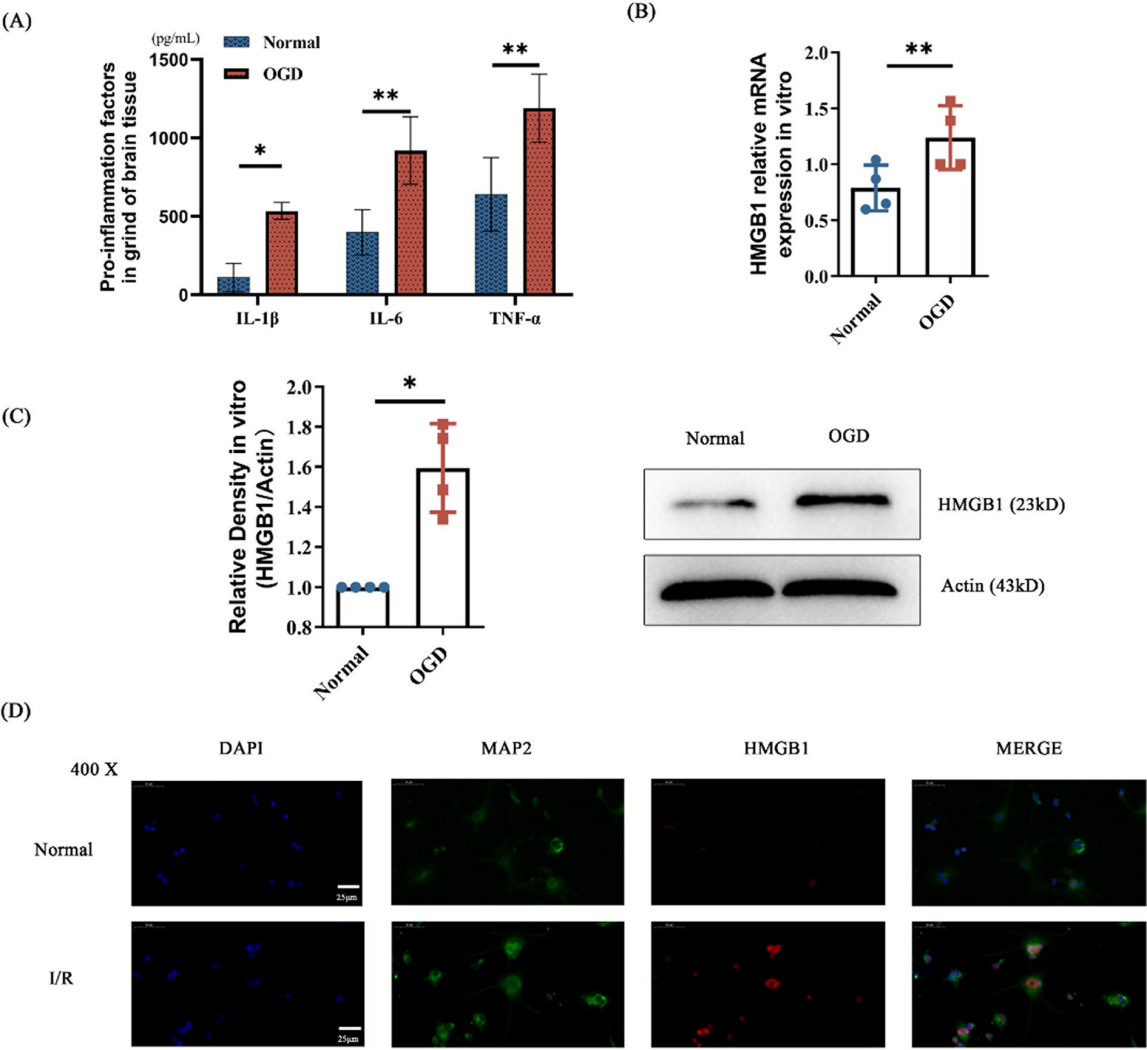
After hypoxia reoxygenation experiments, mouse primary cortical neurons were tested for proinflammatory factors and HMGB 1 expression. **A** Proinflammatory factor levels in primary cortical neurons in control and hypoxic reoxygenation group. **B** HMGB1 mRNA levels in primary cortical neurons in the control and hypoxic reoxygenation groups. **C** The HMGB1 protein levels in primary cortical neurons in control and hypoxia relative to the internal parameters.** D** HMGB1 immunofluorescence levels in primary cortical neurons in control and hypoxia reoxygenation groups. DAPI localized the nucleus and MAP 2 as a neuronal marker. n = 4, * p < 0.05, ** p < 0.01, *** p < 0.001

After verifying the reperfusion damage in vivo as well as HMGB1 expression, we continued to try to explore it in vitro. Primary neurons were extracted from 24-h neonatal mice for subsequent detection. First, we performed oxygen glucose deprivation (OGD) experiments on the cells, and then collected the cell supernatant to detect the expression levels of proinflammatory factors. The results showed that neurons secreted pro-inflammatory factors after OGD (Fig. 2A). The HMGB1 expression level was measured by western blot and Q-PCR assay. HMGB1 level was increased in OGD group in vitro (Fig. 2B-C). Immunofluorescence measurement of neuronal cells also indicated that OGD group had a higher level of HMGB1 (Fig. 2D).

## Silencing of HMGB1 reduces cerebral ischemia–reperfusion injury in mice

**Fig. 3:**
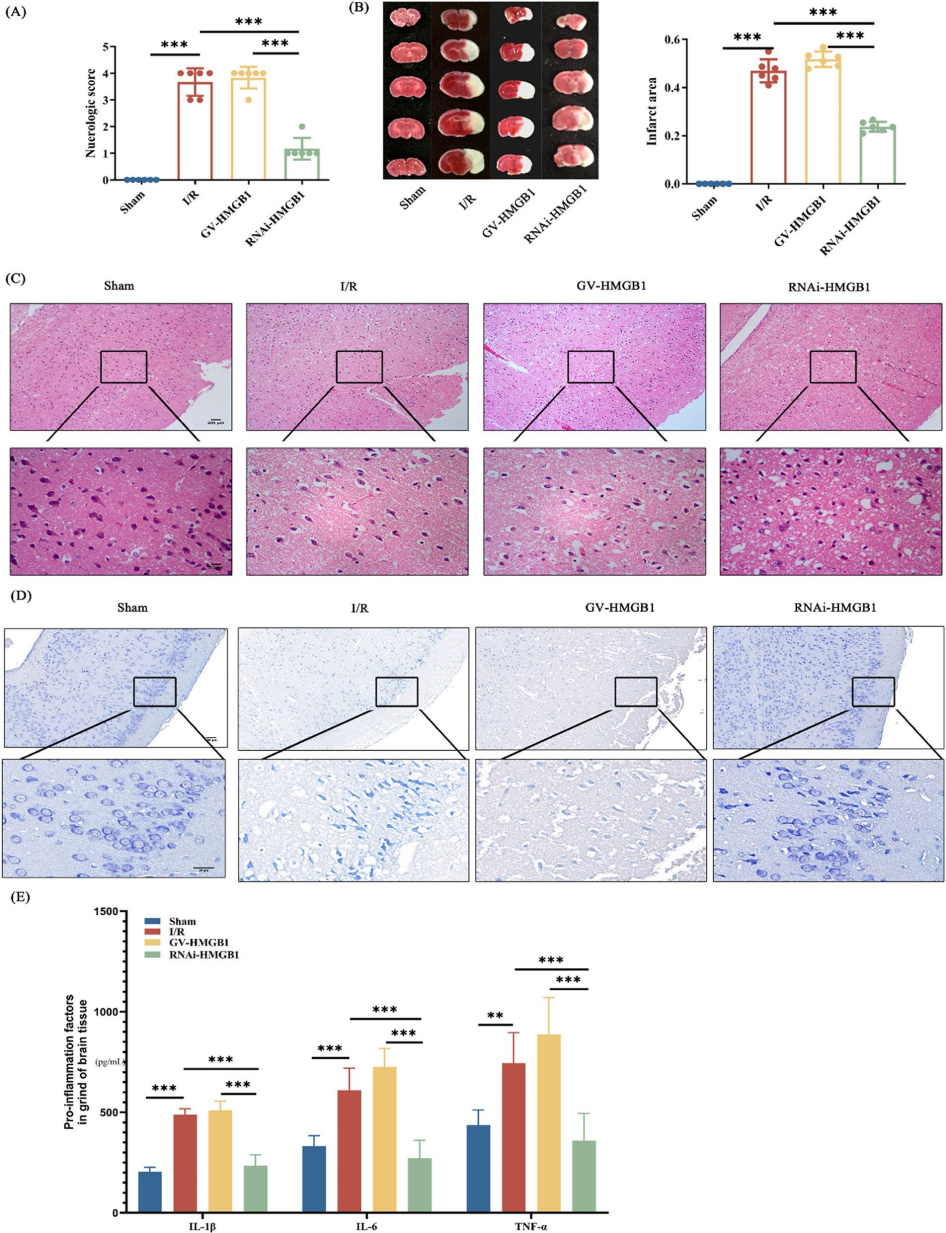
Silencing of HMGB1 reduces cerebral ischemia–reperfusion injury in mice. **A** Neurobehavioral evaluation of mice in different groups. Higher scores indicate more severe neurological damage. **B** TTC staining of serial brain sections of the mice. White represents the infarcted fraction. **C** HE staining of the cortical portion of the mouse brain. Nuclei are colored in blue and the cytoplasm is colored in red. **D** Partial Nic staining of mouse cerebral cortex. The large and large number of Nissl bodies indicates that nerve cells have a strong function in protein synthesis; on the contrary, Nissl bodies will decrease or even disappear when nerve cells are damaged. **E** Detection of proinflammatory factors in ipsilateral brain tissue of control mice and MCAO mice. n = 6, * p < 0.05, ** p < 0.01, *** p < 0.001

To further explore the effect of HMGB1 on inflammation in MCAO mice, we performed overexpression or silencing of HMGB 1 in mice with lateral ventricles. The results showed that after silencing HMGB 1, the neurobehavioral score decreased significantly after cerebral ischemia and reperfusion, suggesting reduced nerve injury in mice (Fig. 3A). More intuitively, in the staining experiment of serial brain sections of mice, mice in the HMGB1 silenced group had a significant reduction in cerebral infarction volume (Fig. 3B). As before, in the HE-stained and Ni-stained sections, we also observed that the brain damage was largely relieved after HMGB1 silencing (Fig. 3C-D). After observing a series of representations, we further examined the proportion of proinflammatory factor expression in extracts from mouse brain homogenates. In the HMGB1 overexpression group, the expression of proinflammatory factors was significantly higher than that in the other groups, while the proinflammatory factor level was significantly decreased in the RNAi-HMGB 1 group (Fig. 3E). These results surface, HMGB1 is one of the key factors in the development of inflammation after ischemia and reperfusion injury in mice.

## Silencing of HMGB1 relieved inflammation in vivo and vitro via RAGE/TLR4

**Fig. 4:**
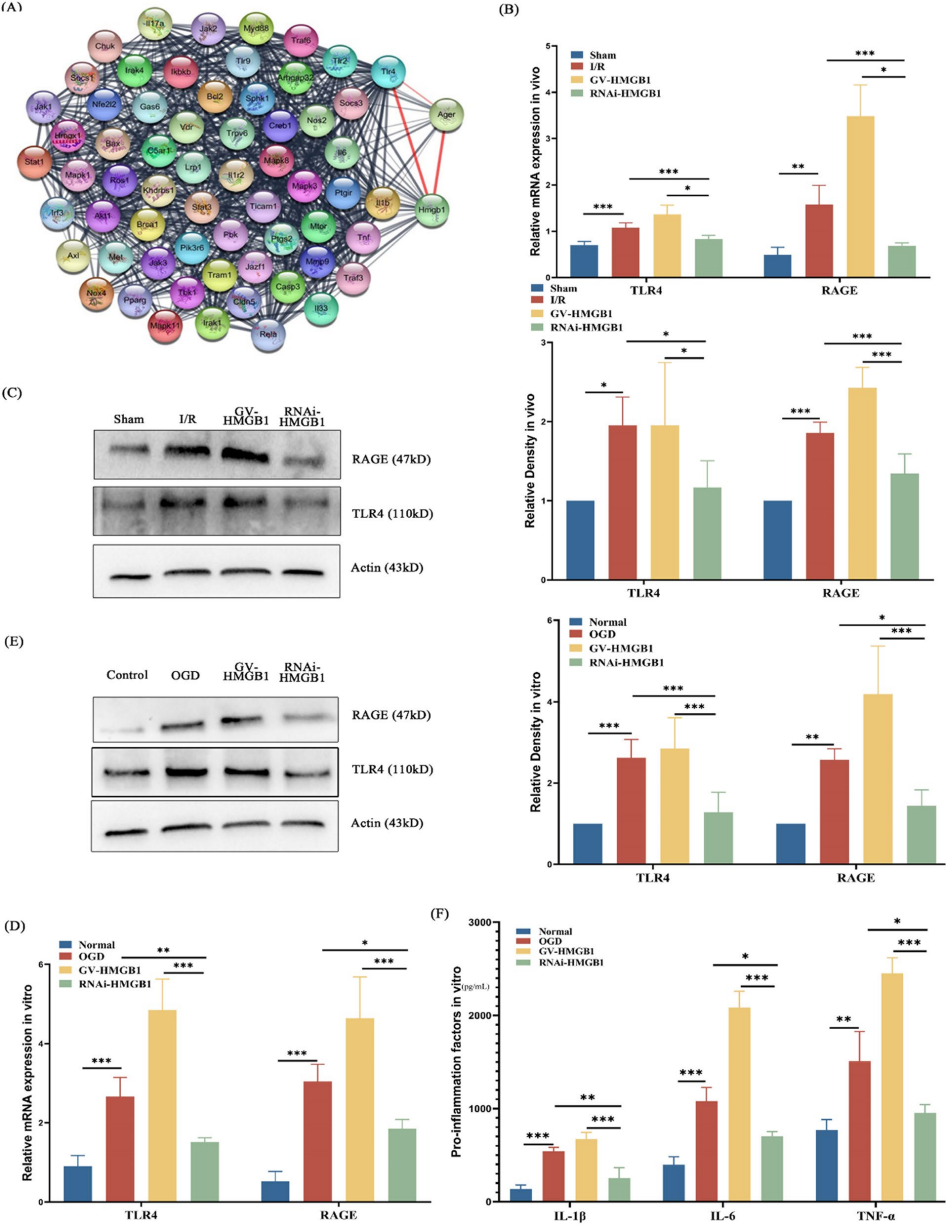
Silencing of HMGB1 relieved inflammation in vivo and vitro via RAGE/TLR4. **A** PPI network in HMGB 1 in inflammatory diseases in the brain. Different colors represent different molecules, and the connecting lines represent the existence of mutual relations. Shorter the connecting line, fewer nodes pass through, indicating stronger interrelationships. **B** HMGB1 mRNA levels in ipsilateral brain tissues in different groups. **C** Protein levels of HMGB1 relative to internal reference in ipsilateral brain tissue from different groups mice. **D** HMGB1 mRNA levels in primary cortical neurons in different groups. **E** The HMGB1 protein levels in primary cortical neurons in different groups. **F** Detection of proinflammatory factors in culture supernatant of primary neuronal cells. n = 6 in vivo experiments and n = 4 in vitro experiments. * p < 0.05, ** p < 0.01, *** p < 0.001

In this part of the study, we sought to further find the mechanism by which HMGB 1 regulates inflammation. We first adopted the network pharmacological induction method and found that RAGE and TLR 4 were the most highly associated with HMGB 1 in inflammatory diseases (Fig. 4A). For further validation, we examined the mRNA and protein content of RAGE and TLR 4 in mouse brain tissues. We found that RAGE and TLR 4 were elevated after injury, and they were highest in injured mice overexpressing HMGB1. Also, evidence that HMGB1 may regulate inflammation through RAGE/TLR4 is that RAGE/TLR4 expression is significantly reduced after silencing HMGB1 in injured mice (Fig. 4B-C). Similarly, this result was again on subsequent primary mouse neurons (Fig. 4D-E). Finally, in this part, we examined the expression of proinflammatory factors in the culture supernatant of mouse primary neuronal cells. What we can know is the fact that overexpression of HMGB1 brings neurons to higher levels of proinflammatory factors after OGD, and silencing of HMGB1 partly reduces proinflammatory factor expression (Fig. 4F). The results of this part basically suggest that HMGB 1 may affect the inflammatory process by regulating RAGE/TLR4 expression and thus affecting the expression of proinflammatory factors.

## BCP protects mice from cerebral ischemia–reperfusion injury

**Fig. 5:**
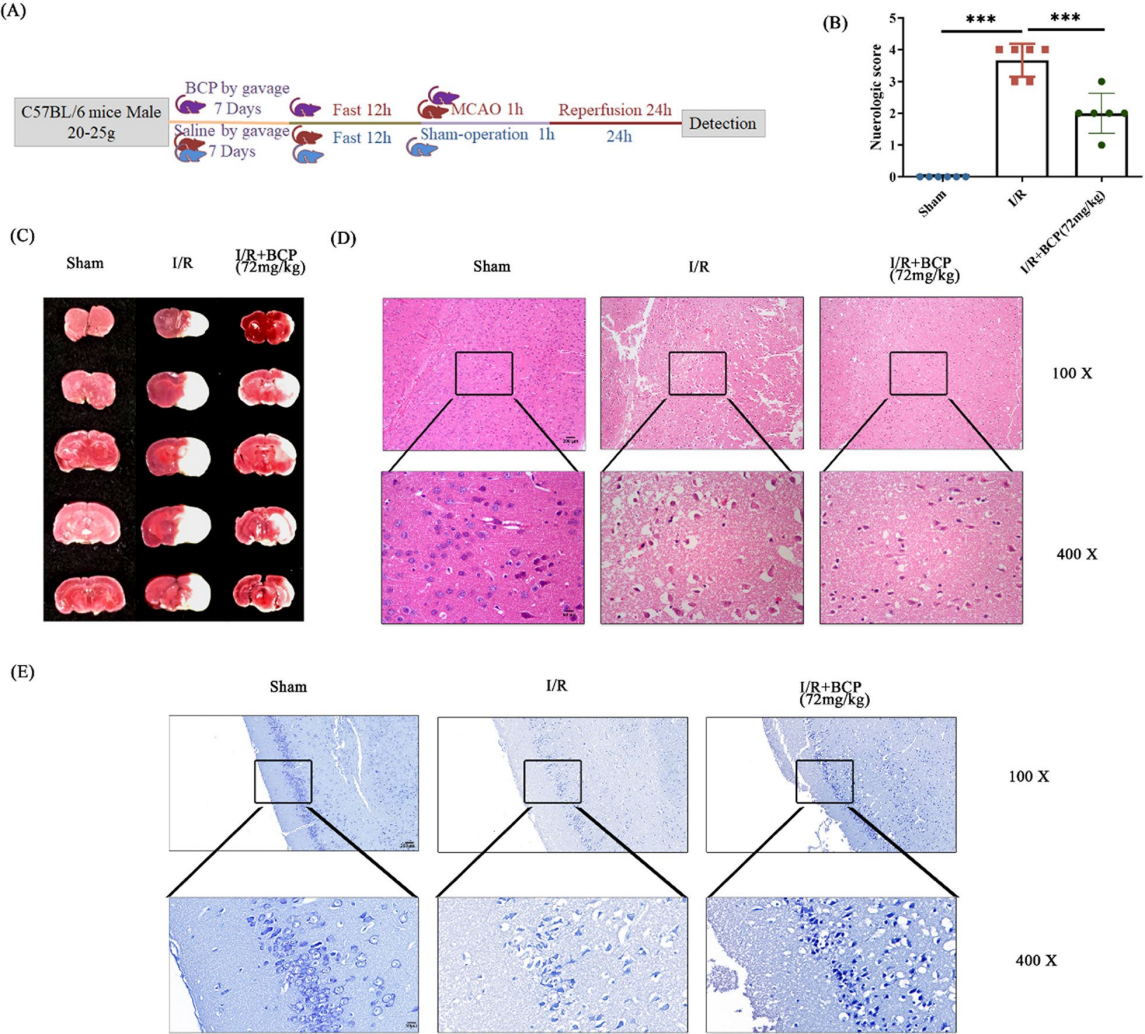
β-caryophyllene can play a protective role in cerebral ischemia and reperfusion. **A** Timeline of the mouse experiments. **B** Neurobehavioral evaluation of mice with or without MCAO. Higher scores indicate more severe neurological damage. **C** TTC staining of serial brain sections of the mice. White represents the infarcted fraction.** D** HE staining of the cortical portion of the mouse brain. Nuclei are colored in blue and the cytoplasm is colored in red. **E** Partial Nic staining of mouse cerebral cortex. The large and large number of Nissl bodies indicates that nerve cells have a strong function in protein synthesis; on the contrary, Nissl bodies will decrease or even disappear when nerve cells are damaged. n = 6, * p < 0.05, ** p < 0.01, *** p < 0.001

It has long been a consensus that drugs to treat brain diseases must be able to cross the blood–brain barrier. Our group found that BCP can cross the blood–brain barrier, and it was reported to have anti-inflammatory effects. In our study, we gave mice 72 mg / kg BCP daily one week earlier and performed MCAO surgery one week later (Fig. 5A). The mice were scored at the experimental endpoint and found that the mice in the BCP group significantly improved stroke (Fig. 5B). Similarly, the cerebral infarction volume was significantly reduced in mice fed with BCP a week earlier after MCAO surgery (Fig. 5C). And the results of Ni staining and HE staining also proved again that BCP has a better neuroprotective effect (Fig. 5D-E).

## BCP protects mice from cerebral ischemia–reperfusion injury by regulating HMGB1

**Fig. 6:**
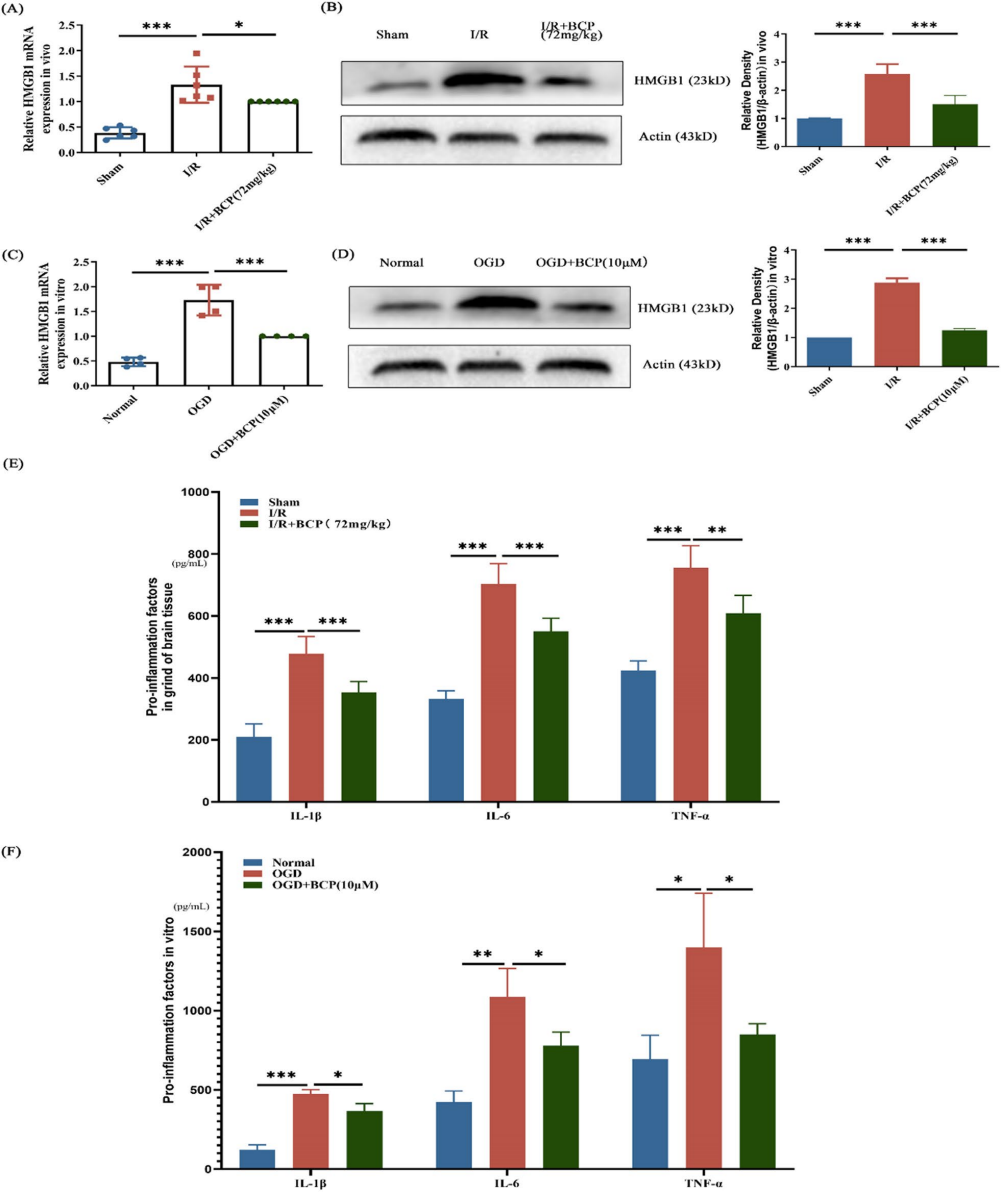
β-caryophyllene play a protective role in cerebral ischemia and reperfusion maybe related to the effect on HMGB1 expression. **A** HMGB1 mRNA levels in ipsilateral brain tissues in different groups. **B** Protein levels of HMGB1 relative to internal reference in ipsilateral brain tissue from different groups mice. **C** HMGB1 mRNA levels in primary cortical neurons in different groups. **D** The HMGB1 protein levels in primary cortical neurons in different groups. **E** Detection of proinflammatory factors in ipsilateral brain tissue of different group mice.** F** Detection of proinflammatory factors in culture supernatant of primary neuronal cells. n = 6 in vivo experiments and n = 4 in vitro experiments. * p < 0.05, ** p < 0.01, *** p < 0.001.

In the previous study, we found that BCP has a promising therapeutic effect of neuroinflammation in mice. BCP has been shown to have anti-inflammatory effects, and HMGB 1 in turn is a key molecule associated with inflammation, so we went on to detect HMGB 1 changes in mouse brain as well as in mouse primary neurons after BCP treatment. We found that both in the mRNA and protein levels, brain HMGB1 levels were significantly lower than those in the BCP group (Fig. 6A, B). This suggests that BCP exerting neuroprotective effects may be related to HMGB1. This result is consistent with the experimental results of primary mouse neurons (Fig. 6C, D). Finally, the anti-inflammatory and protective effects of BCP were also effectively reduced in vivo and in vitro (Fig. 6E, F), which further demonstrated the levels of BCP.

## Discussion

Our study revealed a strong association between HMGB1 expression and stroke incidence. After ischemic stroke, a series of inflammatory reactions occur. Stroke is a disease caused by insufficient or abnormal blood supply to the brain due to sudden obstruction or rupture of cerebral blood vessels(Tu, et al. 2023), and its pathogenesis involves dysregulation of multiple inflammatory responses and neuroprotective mechanisms. As a proinflammatory molecule, HMGB1 can play a role in the development and progression of ischemic stroke. Some studies have shown that cell death occurs with the release of HMGB1, and the role of HMGB1 in necrosis and inflammation has been studied (Kaczmarek, et al. 2013; Scaffidi, et al. 2002; Yanai, et al. 2009). HMGB1 is closely related to inflammation. It is a nuclear protein that can be released from the cell during damage or inflammation and acts as a proinflammatory signaling molecule involved in the regulation of the inflammatory response (Treutiger, et al. 2003). HMGB1 promotes the development and maintenance of the inflammatory response through several mechanisms. Moreover, HMGB1 can also recruit and activate immune cells and increase their activity during inflammatory cell infiltration and tissue damage repair. Therefore, HMGB1 plays an important role in the regulation of inflammation, participating not only in the initiation phase of the inflammatory response but also in the persistence and chronicity of inflammation.

We found that HMGB1 can bind to RAGE and TLR4 and activate downstream inflammatory signaling pathways, leading to the release of inflammatory factors such as TNF-α, IL-1β and IL-6 and triggering an inflammatory response. These results were verified in both animal experiments and cell experiments in primary neurons in our study. To confirm these results, we overexpressed and silenced HMGB1 both in vivo and in vitro. These findings provide strong and direct evidence that high expression of HMGB1 can aggravate nerve injury and inflammation after stroke.

At a time when stroke treatment is in a bottleneck, the development of drugs targeting key factors in the disease is urgently needed. Our findings are important and meaningful; drugs that reduce HMGB1 expression may be potential drugs for the treatment of stroke. An important criterium in the search for drugs for the treatment of brain diseases is their ability to pass through the blood‒brain barrier. BCP is a naturally occurring terpene compound widely found in many plants, especially at high levels in cannabis. It has several biological activities, including anti-inflammatory, antioxidant, antibacterial, and analgesic effects. In pharmacological studies, BCP has been considered to have therapeutic potential for several diseases, including inflammatory diseases, neurodegenerative diseases and pain management. In addition, its antifungal and insect resistance effects on plants have been studied.

Some previous basic science studies by our team support the potential neuroprotective effects of BCP, but its specific application in stroke treatment still needs more clinical experiments and in-depth research to validate its safety and efficacy. Therefore, the current research on the use of BCP for stroke treatment is in its infancy and requires further scientific exploration and validation. We also verified the effect of BCP on HMGB1 expression in vitro and in vivo and found that it could effectively reduce the expression of HMGB1. These findings suggest that the anti-inflammatory and neuroprotective effects of BCP may involve HMGB1.

However, several limitations should be acknowledged. First, the study focused primarily on the HMGB1 pathway, while other potential mechanisms through which BCP exerts its effects were not explored. Future studies should investigate additional molecular pathways and their interactions with HMGB1 to provide a more comprehensive understanding of the neuroprotective properties of BCP. Second, while our results in the mouse model are encouraging, the translational potential of BCP requires validation in larger animal models and, ultimately, in clinical trials. The pharmacokinetics, optimal dosing, and safety profile of BCP in humans remain to be elucidated.

Moreover, this study used in vivo and in vitro models to evaluate the effects of BCP on HMGB1 expression and inflammation. Although these models are well established, they may not fully recapitulate the complexity of human ischemic stroke. Future research should employ advanced techniques such as single-cell sequencing or spatial transcriptomics to reveal the precise cellular targets and mechanisms of BCP.

In conclusion, this study provides a novel perspective on the role of BCP in regulating HMGB1 expression and inflammation, demonstrating its potential for application in stroke treatment. Nonetheless, further investigations are needed to validate these findings and elucidate the broader implications of BCP in neuroprotection.

## Conclusion

In summary, this study confirmed for the first time that BCP may affect the neuroprotective effect of downstream HMGB1 and other inflammatory factors. However, the specific mechanism of BCP needs to be further explored. This study provides a new potential therapeutic target and research direction for the treatment of stroke.

## Data Availability

No datasets were generated or analysed during the current study.

## Abbreviations

I/R: Ischemia/Reperfusion
NF-κB: Nuclear Factor Kappa B
IL: Interleukin
OGD/R: Oxygen–Glucose Deprivation/Reoxygenation
MCAO: Middle Cerebral Artery Occlusion
HMGB1: High-mobility group box-1
RAGE: Receptor for advanced glycation end products
TLR4: Toll-like receptors 4
BCP: β-Caryophyllene
ELISA: Enzyme-Linked Immunosorbent Assay
RT-qPCR: Real-Time Quantitative Polymerase Chain Reaction
TTC: 2,3,5-Triphenyltetrazolium Chloride
RNAi: RNA interference
MAP2: Microtubule-Associated Protein 2
DAPI: 4’,6-Diamidino-2-phenylindole
PBS: Phosphate-Buffered Saline
FBS: Fetal Bovine Serum
DMEM: Dulbecco’s Modified Eagle Medium
EDTA: Ethylenediaminetetraacetic Acid
BCA: Bicinchoninic Acid
PVDF: Polyvinylidene Difluoride
GAPDH: Glyceraldehyde 3-Phosphate Dehydrogenase
SDS-PAGE: Sodium Dodecyl Sulfate Polyacrylamide Gel Electrophoresis
